# Detection of mammagloblin by RT-PCR as a biomarker for lymph node metastasis in breast cancer patients: A systematic review and meta-analysis

**DOI:** 10.1371/journal.pone.0216989

**Published:** 2019-05-23

**Authors:** Ana Monsalve-Lancheros, Milcíades Ibáñez-Pinilla, Sandra Ramírez-Clavijo

**Affiliations:** 1 Faculty of Natural Science and Mathematics, Universidad del Rosario, Bogotá DC, Colombia; 2 School of Medicine and Health Sciences, Universidad del Rosario, Bogotá DC, Colombia; Universita degli Studi di Verona, ITALY

## Abstract

**Background:**

This meta-analysis presents evidence regarding the diagnostic accuracy of mammaglobin detected using the RT-PCR technique, related to the presence of sentinel node metastasis in breast cancer patients.

**Methods:**

The following databases were consulted: Cochrane, Lilacs, Scielo, Hinary, PubMed, Elsevier, Embase, ProQuest, the Universidad del Rosario´s Centro de Recursos Para el Aprendizaje y la Investigación (CRAI-UR) [Resource Center for Learning and Research], and the Google Scholar search engine. The quality of the studies was assessed using the QUADAS-2 and CASpe tools. The selected studies presented the necessary data to calculate diagnostic validity index of mammaglobin detection using RT-PCR, compared with the reference standard test. Global values for the sensitivity, specificity, positive predictive value, negative predictive value, probability ratios, diagnostic ORs, and summary ROC curves of this meta-analysis were obtained using the Meta-DiSc 1.4 program.

**Results:**

Initially, 731 articles were obtained; but only 25 were included in the meta-analysis. Sensitivity was 84% (95% CI: 83% - 86%), and specificity was 92% (95% CI: 91% - 93%). Positive and negative predictive values were 9.26 (95% CI: 6.47–13.26) and 0.17 (95% CI: 0.13–0.23), respectively. The diagnostic OR was 66.34 (95% CI: 42.52–103.52). The predictive area under the sROC curve was 94.78 (Q = 0.8876).

**Conclusions:**

The evaluated diagnostic index showed that the expression of the mammaglobin biomarker has diagnostic prediction for detecting lymph node metastasis in breast cancer patients, when analyzed using RT-PCR, although more than 50% heterogeneity was found.

## Introduction

Breast cancer is a multifactorial disease characterized by increased cellular proliferation in breast tissue [1]. In 2012, 1.67 million new cases were reported worldwide (age-standardized rate per 100,000 population) [2]. This type of cancer is more common in women; according to IARC figures, it causes 324,000 deaths per year in developing countries and 198,000 deaths per year in developed countries [2]. In developing countries, the higher mortality rate is related to the lack of strategies for timely diagnosis, which allows the disease to spread from the area in which the cancer began to another region of the body, what is known as metastasis, and leading in most cases to the patient´s death [1].

Currently, the diagnosis of breast cancer and metastasis is made using histopathological procedure which detects the changes in cell morphology by hematoxylin-eosin (H&E) staining of breast pathology specimens [3]. Also, immunohistochemical (IHC) analyses identify the presence of biomarkers as an integral part of correct diagnosis, where detection of estrogen receptor (ER), progesterone receptor (PR), and HER2/neu is routinely used to provide both prognostic and predictive information necessary for the management of patients with breast cancer [4]. Over time, diagnostic tests have advanced in search of less invasive and more efficient techniques to render diagnoses within a shorter period of time [3,4]. Thus, molecular tests which seek to identify biomarkers have developed dramatically over the last few years [5]. Accordingly, work is being done on the implementation of highly sensitive and specific techniques for diagnosis and follow-up of the response to treatment [6]. The RT-PCR technique has great potential with regard to sensitivity for the detection of biomarkers related to lymph node metastasis in breast cancer patients, including mammaglobin (MGB). However, studies are needed to evaluate its prognostic and diagnostic value [7].

Mammaglobin is a protein expressed in the epithelium of breast tissue, and in other tissues such as the endometrium, sweat glands and salivary glands. The overexpression of this protein is related to the presence of breast cancer [8]. Numerous original studies have been carried out to validate the mammaglobin expression assays to confirm it as a biomarker for the disease and to study its relationship with metastatic breast processes. Some of them showed that it is feasible to use mammaglobin as a biomarker for lymph node metastasis [9–11]. Recently, a systematic review evaluated the clinical effectiveness and cost-effectiveness of One-step nucleic acid amplification (OSNA) and Metasin for the intraoperative analysis of SLN metastases from breast cancer patients. The OSNA test only analyzes cytokeratin 19 (CK19) gene expression [12] while Metasin assay analyzes CK19 and mammaglobin gene expression. Therefore, in our study we focused on the plausibility of intraoperative analysis of the expression of mammaglobin in sentinel lymph node as a biomarker of the presence of metastasis.

In this paper, a systematic literature review and meta-analysis was carried out to evaluate the diagnostic capacity of mammaglobin expression assays for lymph node metastasis in breast cancer patients, using the RT-PCR technique, compared with the usual histopathology/immunohistochemistry tests. We found that some tests in orfer to evaluate mammaglobin gene expression have had different names, such as GeneSearch ^TM^ Breast Lymph Node (BLN) and Metasin assays.

## Methods

### Search strategy

Currently, there are not similar systematic reviews or meta-analysis reported. Therefore, a systematic search was carried out using the Medical Subject Heading (MeSH) terms and databases presented in Table 1. Articles published between January 1999 and April 2019, in which breast cancer patients underwent lymph node biopsy in order to detect the presence of mammaglobin using the RT-PCR technique, were included.

**Table 1:** Search strategy by database.

Search strategy	Database
Mammaglobin, mamaglobina, mamoglobina	Cochrane
(“mammaglobin”) AND (“breast” OR “mammary”) AND (“polymerase chain reaction” OR “PCR” OR “immunologic” OR “immune*” OR “immuno*” OR “lymph node” OR “lymph nodes” OR “metastasis” OR “metastases” OR “metastasization” OR “sentinel” OR “axilla” OR “axillary”)	Lilacs
Mammaglobin	Scielo
Mamaglobina, mamoglobina	Hinary
(“mammaglobin”) AND (“breast cancer” OR “mammary cancer”) AND ("nucleic acid amplification" OR “polymerase chain reaction” OR “PCR”) AND (“immunologic” OR “immune*” OR “immuno*” OR “lymph node” OR “lymph nodes” OR “metastasis” OR “metastases” OR “metastasization” OR “sentinel” OR “axilla” OR “axillary”)	PubMed
mammaglobin AND (breast cancer OR breast neoplasm OR breast tumors) AND (immunological techniques OR immunological marker) AND (polymerase chain reaction OR breast molecular markers OR gene markers) AND (lymph node OR axillary lymph node) Mammaglobin Mammoglobin	Elsevier
mammaglobin AND (breast neoplasm) AND (immune techniques OR immune marker) AND (polymerase chain reaction OR breast molecular markers) AND (lymph node)	CRAI-UR
allintitle OR abstract: mammaglobin+(breast neoplasm)+(immunological marker)+(polymerase chain reaction)+(sentinel lymph node))+epidemiological studies	Google Scholar
(“mammaglobin”) AND (“breast cancer” OR “mammary cancer”) AND ("nucleic acid amplification" OR “polymerase chain reaction” OR “PCR”) AND (“immunologic” OR “immune*” OR “immuno*” OR “lymph node” OR “lymph nodes” OR “metastasis” OR “metastases” OR “metastasization” OR “sentinel” OR “axilla” OR “axillary”)	Embase
(“mammaglobin”) AND (“breast cancer” OR “mammary cancer”) AND ("nucleic acid amplification" OR “polymerase chain reaction” OR “PCR”) AND (“immunologic” OR “immune*” OR “immuno*” OR “lymph node” OR “lymph nodes” OR “metastasis” OR “metastases” OR “metastasization” OR “sentinel” OR “axilla” OR “axillary”) Mammaglobin Mammoglobin	ProQuest

The search strategy was adapted to each database. A comprehensive search could not be performed in all the databases, as the combination of MeSH terms did not always produce any results. Therefore, in some cases, only the keyword ¨mammaglobin¨ was used, since this was the main review term. The EMBASE search results were filtered to avoid those duplicated on PubMed. When duplicate articles were found, they were manually deleted.

### Inclusion and exclusion criteria

An essential requirement for obtaining the data was the construction of 2x2 tables using the results from studies, which mammaglobin was detected in the lymph nodes of breast cancer patients, using RT-PCR tests. The contrast test for the diagnostic evaluation in this case was histopathology (hematoxylin-eosin–H&E), which is the reference test, or the immunohistochemistry as a gold standard probe.

The inclusion criteria were defined as follows:

1. Type of study: prospective and retrospective cohort observational study articles.
2. Patients: individuals with breast cancer who underwent lymph node histopathology to detect metastasis, and in whom the presence of mammaglobin was also detected in the same tissue using the RT-PCR technique. The studies should indicate the patients who were positive or negative for lymph node metastasis by histopathology, and for each of those groups, those who had a positive or negative results of RT-PCR for mammaglobin.
3. Gender: studies carried out on women and also on men, when the researchers included them, but did not disaggregate by gender in their results.
4. Language: studies were analyzed regardless of their language.
5. Time: January 1998 (year of publication of the oldest article in the database search results) to April 2019 (when the last literature search was performed).

The inclusion criteria were established and communicated amongst the involved researchers. The information review stages, article selection and data extraction were carried out independently by two researchers.

### Information quality analysis

This meta-analysis was conducted based on the PRISMA guidelines (S1 Table) and the information quality analysis was performed using the QUADAS-2 and CASpe scales, which allowed choosing those studies with a diagnostic accuracy information that fulfilled the established criteria.

### Data extraction

The data extracted from each study included the patients´ ages, the number of individuals, results of histopathological tests, sentinel lymph node (SLN) or axillary lymph node (ALN) analyses, type of mammaglobin analyzed (since there are two variants of this protein: MGB1 or MGB2), type of RT-PCR carried out and the performance of the test.

### Data required for evaluating lymph node metastasis in breast cancer patients

Metastasis is the term used to describe the ability of the cancer cells to migrate from the site where the lesion began and establish a new lesion in another region of the body.

It is identified as micrometastasis when the dissemination area is less than 0.2 mm or 200 cells, and as macrometastasis when the values are above these reference figures [13].

The presence of malignant cells in the lymph nodes was taken to be indicative of metastasis. The nodes are distributed around the breast; most are directed towards the axillary region and are very important, since the lymph collects the waste products from the extracellular fluid and the immune system cells. In this process, malignant cells may be mobilized [14,15].

Anatomically, the sentinel node is the first regional node to receive lymphatic fluid derived from the mammary gland. Its pathological evaluation is essential in determining the degree of tumor dissemination, since the presence of malignant cells in this structure indicates the development of metastasis. Also, the detection of metastasis in more than one lymph node is valuable information for selecting the most pertinent treatment and evaluating if the axillary lymphadenectomy is required [3,16].

Staining techniques are routinely used for tissue metastasis evaluation, such as hematoxylin-eosin (H&E), which was taken as the gold standard. This technique consists in observing the cellular morphology using a light microscope in order to detect abnormalities. It may be employed together with immunohistochemistry, which uses marked antibodies to detect the presence of proteins such as mammaglobin [3].

The three trials based in qRT-PCR technique were included in order to determine the genes expression. Which was used in the breast lymph node assay (BLN assay) to detect lymph node metastases larger than 0.2 mm. Another assay was the GeneSearch BLN (Veridex LLC, Warren, NJ, USA) which evaluates the mammaglobin and CK19 gene expression in the sentinel nodes of the above-mentioned patients. When the detection of one or both of these markers was considered to be a positive result, axillary emptying was then performed. Although the clinical procedure results were favorable, it was approved by the Food and Drug Administration (FDA), and frequently used in Europe, it was removed from the market due, it seems, to a low consumption of the test in the United States [17,18].

Other groups of researchers experimented with a assay known as Metasin, which is also based on qRT-PCR, to investigate the presence of the two BLN assay markers and a third marker, porphobilinogen deaminase (PBGD), which corresponds to the internal control. A positive result for one or all of the epithelial cell-specific markers and a positive control suggests the presence of metastatic disease in the analyzed lymph node [17,19,20].

### Statistical analysis

This meta-analysis followed the random effects model where the DerSimonian-Laird method was applied to weigh the effect size of the study event in terms of precision, with precision being directly related to each study´s sample size and variability. The publication bias was evaluated using a funnel plot, and homogeneity between studies was evaluated with the Higgins Index (I^2^). The MetaDiSc 1.4 software was used to obtain the necessary statistics for interpreting the meta-analysis, as well as the forest plots of the pooled measures of sensitivity, specificity, PPV, NPV, diagnostic OR and sROC curve with their standard errors and 95% confidence intervals. The intra and inter-study heterogeneity visible in the forest plots was also evaluated [21]. In addition, a subgroup analysis was performed to show the behavior of the analyzed mammaglobin (MGB1 or MGB2), the type of node (SLN or ALN), and the type of PCR technique (RT-PCR, qRT-PCR or BLN assay).

## Results

### Study selection

**Fig 1:**
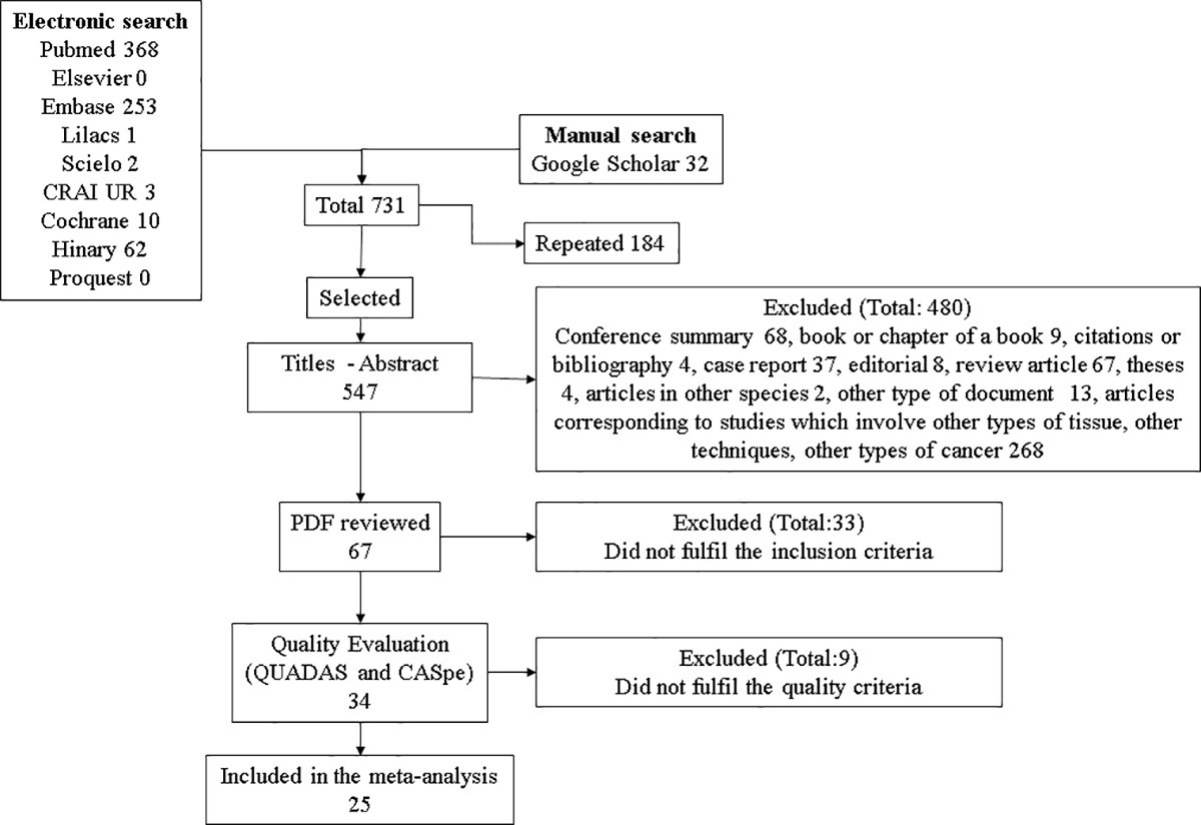
Article selection diagram. A detailed description of the article selection.

Out of a total of 264 studies obtained from the databases, 24 was remained after applying the inclusion and exclusion criteria and quality tests, and were included in this meta-analysis. Of these, 22 were found in the PubMed database and 2 in Google Scholar. Twenty-two of the 24 were published in English, one in Portuguese, and one in Mandarin. Fig 1 shows the article selection scheme. A table was constructed with the data provided by the various articles, which is shown in S1 File.

The following are the references of the articles included in this study: Smith et al (2017) [20], Sai Giridhar et al (2016) [19], Ramadhani et al (2013) [17], Wallwiener et al (2011) [22], Funasako et al (2010) [23], Li et al (2010) [24], Mansel et al (2009) [10], Martínez et al (2009) [18], Veys et al (2009) [9], Julian et al (2008) [11], Viale et al (2008) [25], Berger et al (2006) [26], Brown et al (2006) [27], Dell’Orto et al (2006) [28], Nissan et al (2006) [7], Backus et al (2005) [29], Menezes et al (2005) [30], Ouellette et al (2004) [8], Branagan et al (2002) [31], Manzotti et al (2001) [32], Marchetti et al (2001) [33], Kataoka et al (2000) [34], Ooka et al (2000) [35], Tafra et al (2000) [36], Aihara et al (1999) [37].

### Information quality assessment

**Fig 2:**
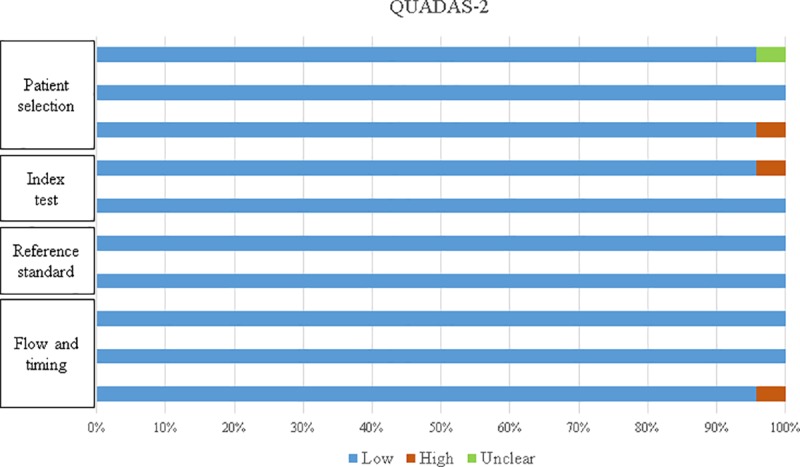
QUADAS scale. Summary of the quality evaluation of the studies according to the QUADAS-2 scale criteria.

According to the results of the QUADAS scale application, the studies generally fulfilled the evaluated criteria. As shown in Fig 2, the risk of bias in the analyzed articles was low.

QUADAS inquiries about the evaluation of the test results independently from the reference standard result. Twenty-three of the 24 studies state that there was independence in the analysis, with the exception of the study by Backus et al. (2005). However, the studies by Sai Giridhar et al. (2016), Mansel et al. (2009), Martínez et al. (2009) and Julian et al. (2008) affirm blind evaluation of the results.

### Publication bias

**Fig 3:**
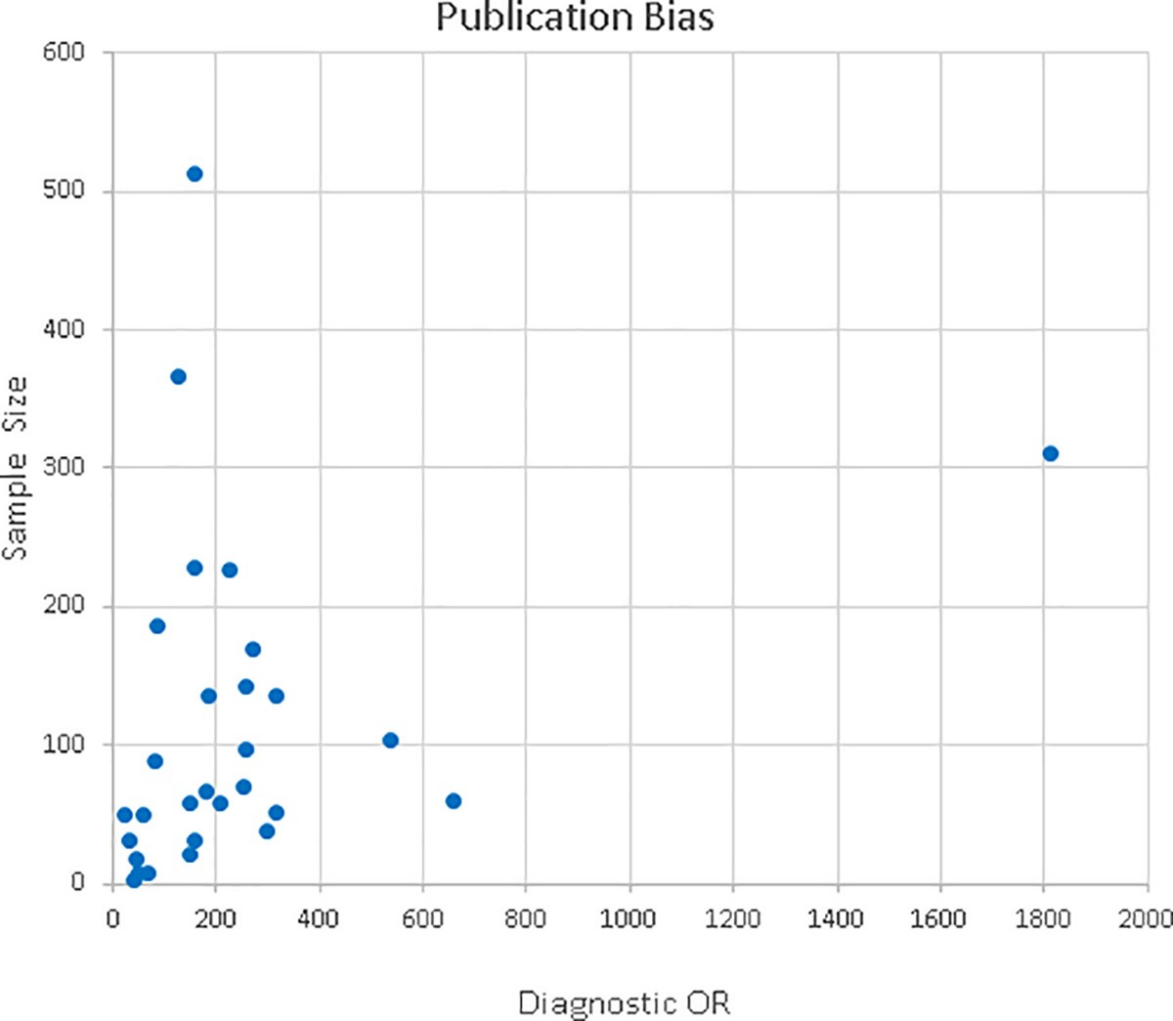
Funnel plot. Publication bias.

This was evaluated by constructing a funnel plot Fig 3. Diagnostic OR values (x-axis) were used against the sample size (y-axis) [38]. The figure shows a funnel-shaped distribution of the data, which allowed us to conclude that there was no tendency towards publishing the studies with the best results (in which case the data would have been located to the right of this graph).

### Diagnostic indices

**Fig 4:**
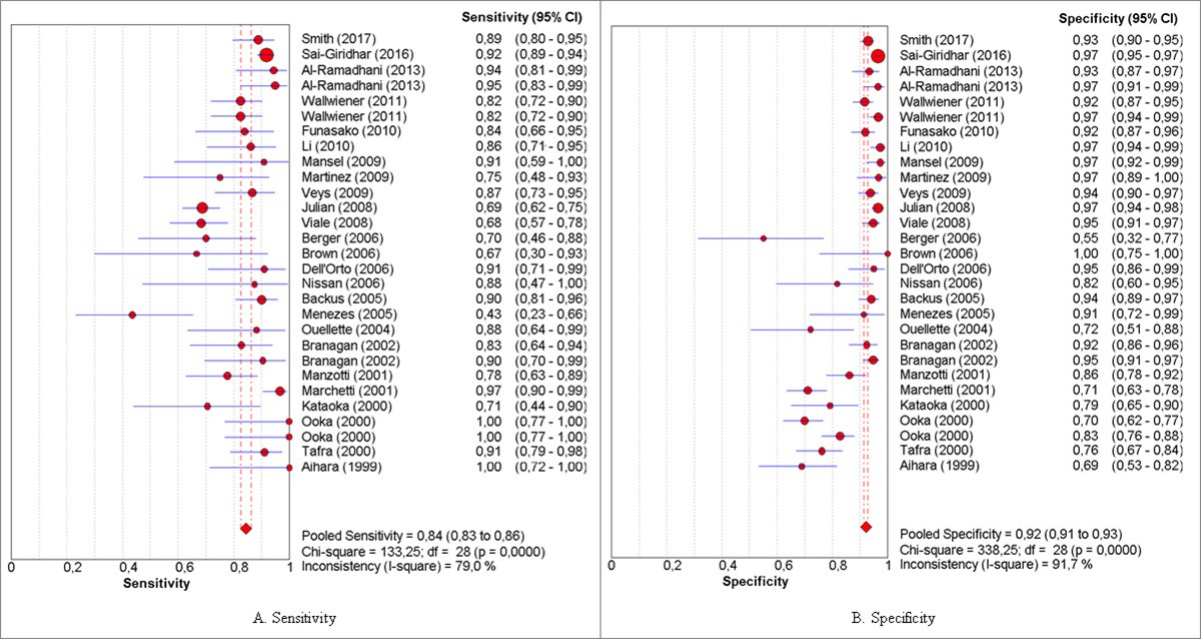
Forest plots of the diagnostic estimators. (A) and (B) show, respectively, the global measures of sensitivity and specificity of the mammaglobin biomarker for breast cancer metastasis.

**Fig 5:**
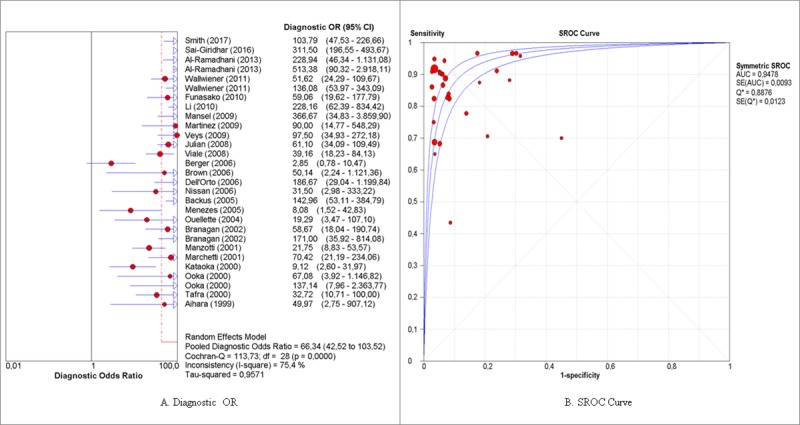
Forest plots of the diagnostic estimators and sROC curve. (A) and (B) show, respectively, the diagnostic OR and sROC curve, which indicate the global performance of mammaglobin for detecting metastasis in breast cancer patients.

Pooled sensitivity, specificity, positive and negative probability, diagnostic OR and sROC curve were obtained with the MetaDiSc 1.4 program. The global sensitivity and specificity obtained were 85%, CI (83%-87%) and 92% CI (91%-93%), respectively Fig 4, and the value of the diagnostic OR was 66.34 CI (42.52%-103.52%) and the sROC curve was 94.77% (Q = 0.8874; SE = 0.0099) Fig 5.

### Subgroup analysis

A subgroup analysis was performed considering the variables: sentinel or axillary lymph node tests, type of mammaglobin, and variants of the RT-PCR technique. However, not all authors reported the type of mammaglobin analyzed (S1 File), and therefore the analysis was only performed for the type of node and RT-PCR variant variables. In this case, the results were divided into two categories: 13 studies which performed sentinel node (SLN) analysis, and six with axillary node (ALN) analysis. The results are shown in Table 2.

**Table 2:** Subgroup analysis according to type of node analyzed.

Node / Pooled measure	SLN				ALN			
		CI	I^2^ (%)	P		CI	I^2^ (%)	P
**Sensitivity**	84%	82%-86%	75.2	<0.0001	87%	81%-91%	86.5	<0.0001
**Specificity**	94%	94%-95%	82.8	<0.0001	81%	78%-83%	91.1	<0.0001
**Positive Likelihood ratio**	11.94	8.52–16.74	86.6	<0.0001	4.25	2.78–6.51	85.7	<0.0001
**Negative Likelihood ratio**	0.17	0.12–0.22	78.3	<0.0001	0.16	0.05–0.52	90.3	<0.0001
**Diagnostic OR**	79.2	50.39–124.49	74.3	<0.0001	34.92	10.01–121.7	70.1%	<0.0001
**sROC curve**	AUC = 0.9495 Q = 0.8899 SE(Q) = 0.0147				AUC = 0.9162 Q = 0.8490 SE(Q) = 0.0405			

Additionally, Table 3 shows subgroup analysis variability due to variants of the RT-PCR technique, which were used for detecting mammaglobin gene expression. Eleven studies were found to have used qualitative RT-PCR, eight groups used the BLN assay; only four studies using the qRT-PCR were found, and three used the Metasin test. Therefore, these last two subgroups (qRT-PCR and Metasin) data were not shown in Table 3, those can be consulted in S2 File.

**Table 3:** Subgroup analysis according to the type of technique used to detect mammaglobin in the node.

Technique / Pooled measure	RT-PCR				BLN assay			
	Value	CI	I^2^ (%)	P	Value	CI	I^2^ (%)	P
Sensitivity	86%	82%-89%	78.1	<0.0001	76%	71%-80%	71.5	0.0009
Specificity	82%	80%-84%	86.4	<0.0001	95%	94%-96%	37.4	0.1312
Positive Likelihood ratio	4.80	3.63–6.36	76.4	<0.0001	16.03	12.25–20.98	21.8	0.2565
Negative Likelihood ratio	0.17	0.09–0.32	81.2	<0.0001	0.21	0.15–0.31	63.8	0.0072
Diagnostic OR	33.75	20.29–56.13	25.7	0.1843	81.57	50.93–130.63	31.1	0.1798
sROC Curve	AUC = 0.9178 Q = 0.8509 SE(Q) = 0.0166				AUC = 0.9717 Q = 0.9226 SE(Q) = 0.0166			

The studies in which qRT-PCR was performed reported Ct values between 26 and 31 [(26,28,29)], including the BLN kit [9,10,17,18,24,25] and Metasin [17,19,20] assays, except for Funasako et al. (2010) [23] and Julian et al. [11], who reported having followed the commercial protocol criteria, without revealing the details of the protocol. Meanwhile, the studies which evaluated mammaglobin detection using qualitative RT-PCR reported the size of their products, which is related to the type of mammaglobin (MGB1 or MGB2). They also indicated the number of cycles, according to the protocol used; this corresponded to 40 cycles, according to their reports [7,8,27,31–33,35,36], with the exception of Menezes (2005) who reported 46 cycles [30]. On the other hand, Kataoka et al. (2000) and Aihara et al. (1999) indicated the size of their PCR products but did not report the number of cycles used [34,37].

We performed another subgroup analysis, in order to check how each study confirmed the presence or absence of cancer cells, by frozen examination with H&E or (IHC) techniques as a gold standard, data are shown in Table 4

**Table 4:** Subgroup analysis according to a gold standard probe used to detect metastasis in node.

Technique / Pooled measure	H&E				IHC			
	Value	CI	I^2^ (%)	P	Value	CI	I^2^ (%)	P
Sensitivity	81%	78%-83%	77.3	<0.0001	91%	88%-93%	56.0	0.0339
Specificity	91%	90%-92%	90.4	<0.0001	95%	94%-96%	93.0	<0.0001
Positive Likelihood ratio	8.73	6.15–12.38	90.4	<0.0001	11.37	3.98–32.45	95.8	<0.0001
Negative Likelihood ratio	0.19	0.14–0.26	75.1	<0.0001	0.15	0.07–0.29	63.86	<0.0001
Diagnostic OR	59.71	42.20–84.49	43.6	0.0157	88.93	21.09–375.06	89.8	<0.0001
sROC Curve	AUC = 0.9468 Q = 0.8862 SE(Q) = 0.0109				AUC = 0.9509 Q = 0.8917 SE(Q) = 0.0345			

## Discussion

### Data collection

This meta-analysis is a pioneer in data compilation and the analysis of evidence contributing information regarding studies that allow the evaluation of the capacity of mammaglobin detection, using the RT-PCR technique, as a biomarker for lymph node metastasis in breast cancer patients.

In order to provide a wide possibility of inclusion of scientific publications in this review, various search terms were used, in accord with the MeSH nomenclature, and the terms were searched for in both the titles as well as the abstracts. As a result, 264 publications were found, and after applying the inclusion and exclusion criteria as well as the quality scales, 24 articles were included in the meta-analysis.

Gathering the studies that sufficiently fulfill the criteria for a meta-analysis is a complex task. Liu et al. (2014) included 42 studies (beginning with 2,243 studies found on PubMed and Embase) to analyze the CEA molecule and 10 for CA242, as colorectal cancer biomarkers. Madeira et al. (2015) included 12 studies (the search was carried out on the MEDLINE, Embase, Cochrane, IBECS, BIOSIS, Web of Science, SCOPUS, and Congress Abstracts databases, as well as on Google Scholar and The British Library, where 111 studies were found) in a meta-analysis to evaluate the mesothelin molecule as a biomarker in ovarian cancer [39,40]. Consequently, 24 articles which fulfilled a set of criteria and conditions for evaluating the available evidence were analyzed in order to answer this study´s research question.

### Publication bias

The clustering of the data on the funnel plot showed no evidence of publication bias. With regard to the sample sizes of the various studies, some were noted to have small sample sizes, such as, for instance, Brown´s (2006) and Nissan´s (2006) studies in which 22 and 30 subjects were evaluated, respectively.

### Heterogeneity

It is common to find high heterogeneity in meta-analysis, due to the effect of different factors. It should be kept in mind that the studies included in this review have highly variable sample sizes, which could have had an effect on the elevated I^2^ values obtained in this meta-analysis´ global measures. The study with the smallest sample size was Brown´s (2006), with 22 individuals. The largest sample size was contributed by the studies performed by Sai Giridhar (2016) and Julian (2008), with 1,809 and 656 study subjects, respectively.

Additionally, a clear fact that leads to heterogeneity in our study is the presence of small observation values in the 2x2 table, which increases the standard errors and therefore produces wide confidence intervals for the diagnostic precision measures.

A classification bias which may influence the measurements is the presence of the threshold effect, caused by the difference in the nature of the qualitative and quantitative RT-PCR techniques. Of the 24 studies analyzed, 11 used qualitative RT-PCR. These studies do not report a threshold value, per se, due to the intrinsic properties of the technique; rather, the protocol is stated and in eight of the published studies, the product obtained was reported, employing 40 cycles [7,8,27,31–33,35,36].

In the case of quantitative RT-PCR, the technique´s cut-off points are related to the difference in Ct values, understood as the PCR cycle in which the fluorescence emitted by the reaction surpasses the fixed threshold. Each protocol requires the construction of a standard curve which allows the number of PCR cycles needed to define when a test is positive or negative to be determined. Thirteen studies used qRT-PCR, including three using the Metasin technique and eight using the BLN assay. These studies reported the threshold value employed for the qRT-PCR technique; a narrow range was reported with a minimum of 26 and a maximum of 31.7 cycles, which would control this type of bias [9,10,16–19,22–24,26, 27].

It may be affirmed that the RT-PCR technique is a robust technique which uses protocols that may differ slightly in their conditions, but which ultimately do not produce significant variations in the amplification results for a given molecule. Thus, the high variability obtained in the different measures for these data indicates that the results should be interpreted cautiously. However, the pooled evidence reveals that the performance of mammaglobin in the detection of lymph node metastasis in breast cancer patients, using the RT-PCR technique, is clinically relevant. The global measure of effect, considering the diagnostic OR, was 64.95, 95% CI: (39.86–105.84), indicating that mammaglobin as a biomarker has a high capacity for diagnostic accuracy of lymph node metastasis in breast cancer patients, according to the stipulated parameters.

### Meta-analysis

The global sensitivity and specificity values (85% and 92%, respectively) indicate the high capacity of mammaglobin to signal the presence of lymph node metastasis.

Heterogeneity can be seen in the positive probability results, since the positive predictive values range from 1.56 to 34.24 (values which were obtained by Berger (2006) and Mansel (2009), respectively). These data illustrate the variability, but it should be emphasized that all of the studies are located on the right of the unit, indicating that the mammaglobin molecule is able to detect those metastases that are truly positive.

For negative probability, the forest plot shows that the included studies are found to the left of the unit, evidencing that mammaglobin is able to detect those lymph nodes which are free from metastasis.

A consideration of the global measure (dOR) shows high intra-study heterogeneity, taking into account the width of the confidence intervals obtained. Of note is the tendency of the results to favor the differentiation of individuals with lymph node metastasis, employing the RT-PCR assay for mammaglobin detection.

When these results are compared with those of other biomarker meta-analysis, the global OR value (66.34 with a CI (42.52–103.52); Q = 113.73; p value<0.0001) may be said to be significant. The Ruiz et al. (2010) group, who evaluated the detection of the PCA3 molecule in the diagnosis of prostate cancer, obtained a global diagnostic OR value of 5.59 (CI (3.96–7.89); Q = 57.30; p<0.0001). Additionally, Madeira et al. (2015) reported a global diagnostic OR of 38.92 (95% CI (17.82–84.99); Q = 41.2, p = 0.0001; I^2^ = 73.3%). These results are similar to those obtained in the current meta-analysis in as much as their OR value is high and statistically significant, although the confidence interval was substantially wide [40,41]

With regard to the sROC curve, in this study the area under the curve was 94.78%. The Q measure of this plot allows an interpretation of the global efficacy of the test, where the values over 0.5 are significant. In addition, the Q value obtained was 0.8876, which is in line with the rest of the indices obtained and corroborates the high efficacy of mammaglobin for detecting lymph node metastasis in breast cancer patients, using the RT-PCR technique.

In addition, this study progressed to the evaluation of subgroups for the variables of lymph node examined and technique used for the analysis. The results of the node variable indicated that mammaglobin may have a greater probability of metastasis detection in the sentinel node; the diagnostic OR values and area under the sROC curve are greater in the sentinel node analysis when compared with the axillary nodes.

With regard to the techniques evaluated in the subgroups, it can be said that this technique shows high sensitivity and specificity for its purpose [4]. Although the analyzed techniques show a remarkable performance in detecting lymph node metastasis, it must be kept in mind that the gold standard tests for this event continue to be immunohistochemistry and histopathology (H&E). It is important to note that lymph node tissue samples analyzed by H&E or IHQ are different from those analyzed by molecular gene expression assays, which may explain the non-concordance of results, in some cases, between these two tests. However, the RT-PCR technique is widely used today in molecular biology for gene expression studies. The protocols should be standardized for each particular case, but it is a technique with a very robust diagnostic performance in detecting mammaglobin, which allows the identification of metastatic processes in the sentinel nodes of breast cancer patients.

The studies carried out with the commercial BLN and Metasin assays showed similar concordance with the gold standard, as reported by the studies in which these techniques were analyzed. According to the analyses performed by Mansel et al. (2009), the concordance between the BLN assay and histopathology was 93.9% [10]. Furthermore, the concordance of the Metasin technique with the reference standard was 94.1%, according to Smith et al.´s (2017) data [20] and 92.3% according to Sai Giridhar´s (2016) findings [19]. However, the BLN assay was discontinued, according to Al-Ramadhani et al.´s (2013) report, by order of the FDA due to low kit use, in spite of having shown positive results in its diagnostic usefulness. In addition, the Metasin technique provides information in a short span of time; 32 minutes according to Al-Ramadhani et al.´s (2013) study and 42 as shown by Smith et al. (2016) [17,20].

It is pertinent to highlight mammaglobin´s contribution as a biomarker for sentinel node metastasis, as shown in this study. Biomarkers are being widely studied due to their relevance for early diagnosis, prognosis and therapeutic decision making in diseases as serious as cancer. Although, as has been stated, studies related to biomarkers are subject to high variability (shown in the forest plots), they are very relevant due to the valuable information they can contribute. This is ratified by the results obtained in this paper and others, such as the meta-analysis proposed by Ruiz and Márquez (2010), who evaluated the detection of the PCA3 gene product through antigen detection in urine samples, as a biomarker for prostate cancer. This group of researchers reported heterogeneity in global sensitivity and specificity of 63% and 74%, respectively, and reported an area under the curve of 0.73 [41].

Liu Z et al. (2014) carried out a meta-analysis to establish the evidence with regard to the accuracy and precision of diagnostic tests in colorectal cancer and reported heterogeneity. Potential sources of variability were reported to be the severity of the illness, age, and sex; and, in the clinical context, the dosage, timing or duration of treatment [39].

Together, the meta-analysis show that the evidence contributed in the analysis of biomarkers is susceptible to significant variations which make its clinical interpretation difficult. However, it may be inferred that the contributions of these meta-analysis can provide great advances which may offer an interpretation and clinical guidance, according to the function performed by the biomarker.

Considering the validity of mammaglobin detection ascertained in this study, a fundamental contribution of these findings to clinical practice is the possibility of obtaining quick results with high sensitivity and specificity for detecting lymph node metastasis in breast cancer patients. Some of the analyzed studies were carried out while the patients were in surgery; thus, the detection of mammaglobin by RT-PCR provides a decision-making tool for the specialist during the surgical procedure. It allows intraoperative node analysis so that, if the findings are positive, axillary emptying may be carried out or the affected nodes removed. This keeps the patient from having to undergo another surgical procedure later on, with its respective preparation and recovery; contributes to improving the disease prognosis, gaining valuable time for the patient in disease progression; and helps lower the procedure´s costs. Thus priority should be placed on development new intraoperative tests under different protocols with better performance and new studies to evaluate the clinical effectiveness and cost-effectiveness that consider the node-positive rate, tissue allocation bias (TAB), lifetime discounted and a quality-adjusted life-year (QALY), similar to Huxley’s et al study [12]. Likewise, criteria related to the size of the Extranodal Extension (ENE) on sentinel lymph node dissection recommended by the American College of Surgeons Oncology Group Z0011 Trial Era can be accepted. These, together with molecular test results, can help to better define the group of patients will undergo a new axillary surgery or be treated with axillary radiotherapy (ART) as an alternative to axillary lymph node dissection (ALND) [42].

## Conclusions

The analyzed indices: sensitivity, specificity, positive and negative predictive values, diagnostic OR and sROC curve, indicated that mammaglobin expression has diagnostic precision for detecting lymph node metastasis in breast cancer patients when it is detected using RT-PCR.

The results presented in this meta-analysis have a favorable clinical impact on patient treatment and disease prognosis. However, it is important to point out the difficulty of the heterogeneity of the studies.

Future studies can supply information to confirm the consistency of this meta-analysis and it may be affirmed that the RT-PCR technique is robust and rapid, applicable in the detection of biomarkers with diagnostic value, such as mammaglobin.

## Supporting information

S1 File Meta-analysis additional data. This file contains the information gathered from the articles about the general characteristics, as well as the detailed table of the meta-analysis and CASpe analyses. Data are presented on separate sheets.
(XLSX)

S2 File Subgroups qRT-PCR and Metasin. This file contains the information about the meta-analysis performed for qRT-PCR and Metasin methods Subgroups.
(RTF)

S1 Table PRISMA 2009 checklist. (DOC)

S1 Fig Prisma 2009 flow diagram. (TIF)
