# Supplementary material for: Detection of mammagloblin by RT-PCR as a biomarker for lymph node metastasis in breast cancer patients: A systematic review and meta-analysis
Source: PLoS One. 2019 May 23;14(5):e0216989. doi: 10.1371/journal.pone.0216989 (PMC6532868; doi:10.1371/journal.pone.0216989)
Supplement: S2 File — This file contains the information about the meta-analysis performed for qRT-PCR and Metasin methods Subgroups. (RTF) [file pone.0216989.s002.rtf]

Method – qRT-PCR

Summary Sensitivity

               Study     |	Sen	[95%  Conf. Iterval.]		TP/(TP+FN)  TN/(TN+FP)
--------------------------------------------------------------------------------------------
Wallwiener (2011)        |	0,824     0,718   - 0,903   		61/74	220/240
Wallwiener (2011)        |	0,824     0,718   - 0,903   		61/74	232/240
Berger (2006)            |	0,700     0,457   - 0,881   		14/20	11/20
Dell'Orto (2006)         |	0,909     0,708   - 0,989   		20/22	56/59
Backus (2005)            |	0,901     0,807   - 0,959   		64/71	172/183
--------------------------------------------------------------------------------------------
          Pooled Sen     |	0,843     0,793   - 0,885   
--------------------------------------------------------------------------------------------
Heterogeneity chi-squared = 5,84 (d.f.= 4) p = 0,212
Inconsistency (I-square) = 31,5 %
No. studies =  5.
Filter ON (Method = 2 )
Add 1/2 to all cells of the studies with zero 


Summary Specificity

               Study     |	Spe	[95%  Conf. Iterval.]		TP/(TP+FN)  TN/(TN+FP)
--------------------------------------------------------------------------------------------
Wallwiener (2011)        |	0,917     0,874   - 0,948   		61/74	220/240
Wallwiener (2011)        |	0,967     0,935   - 0,986   		61/74	232/240
Berger (2006)            |	0,550     0,315   - 0,769   		14/20	11/20
Dell'Orto (2006)         |	0,949     0,859   - 0,989   		20/22	56/59
Backus (2005)            |	0,940     0,895   - 0,970   		64/71	172/183
--------------------------------------------------------------------------------------------
          Pooled Spe     |	0,931     0,911   - 0,948   
--------------------------------------------------------------------------------------------
Heterogeneity chi-squared = 29,26 (d.f.= 4) p = 0,000
Inconsistency (I-square) = 86,3 %
No. studies =  5.
Filter ON (Method = 2 )
Add 1/2 to all cells of the studies with zero 


Summary Positive Likelihood Ratio (Random effects model)

               Study     |	LR+   	[95%  Conf. Iterval.]		% Weight
--------------------------------------------------------------------------------------------
Wallwiener (2011)        |	9,892     6,418   - 15,246  		21,25
Wallwiener (2011)        |	24,730    12,412  - 49,273  		20,04
Berger (2006)            |	1,556     0,886   - 2,732   		20,68
Dell'Orto (2006)         |	17,879    5,890   - 54,269  		17,42
Backus (2005)            |	14,996    8,413   - 26,732  		20,61
--------------------------------------------------------------------------------------------
 (REM) pooled LR+        |	9,791     3,586   - 26,737  
--------------------------------------------------------------------------------------------
Heterogeneity chi-squared = 53,07 (d.f.= 4) p = 0,000
Inconsistency (I-square) = 92,5 %
Estimate of between-study variance (Tau-squared) = 1,1874  
No. studies =  5.
Filter ON (Method = 2 )
Add 1/2 to all cells of the studies with zero 


Summary Negative Likelihood Ratio (Random effects model)

               Study     |	LR-   	[95%  Conf. Iterval.]		% Weight
--------------------------------------------------------------------------------------------
Wallwiener (2011)        |	0,192     0,117   - 0,314   		25,14
Wallwiener (2011)        |	0,182     0,111   - 0,298   		25,16
Berger (2006)            |	0,545     0,251   - 1,188   		18,84
Dell'Orto (2006)         |	0,096     0,026   - 0,360   		10,49
Backus (2005)            |	0,105     0,052   - 0,212   		20,39
--------------------------------------------------------------------------------------------
 (REM) pooled LR-        |	0,189     0,113   - 0,319   
--------------------------------------------------------------------------------------------
Heterogeneity chi-squared = 11,61 (d.f.= 4) p = 0,020
Inconsistency (I-square) = 65,6 %
Estimate of between-study variance (Tau-squared) = 0,2166  
No. studies =  5.
Filter ON (Method = 2 )
Add 1/2 to all cells of the studies with zero 


Summary Diagnostic Odds Ratio (Random effects model)

               Study     |	DOR   	[95%  Conf. Iterval.]		% Weight
--------------------------------------------------------------------------------------------
Wallwiener (2011)        |	51,615    24,292  - 109,67  		22,19
Wallwiener (2011)        |	136,08    53,971  - 343,09  		21,38
Berger (2006)            |	2,852     0,777   - 10,467  		19,33
Dell'Orto (2006)         |	186,67    29,041  - 1199,8  		16,05
Backus (2005)            |	142,96    53,114  - 384,79  		21,05
--------------------------------------------------------------------------------------------
 (REM) pooled DOR        |	55,270    15,130  - 201,90  
--------------------------------------------------------------------------------------------
Heterogeneity chi-squared = 29,18 (d.f.= 4) p = 0,000
Inconsistency (I-square) = 86,3 %
Estimate of between-study variance (Tau-squared) = 1,8208  
No. studies =  5.
Filter ON (Method = 2 )
Add 1/2 to all cells of the studies with zero 


-------------------------------------------------------------------------------------------

Method - Metasin


Summary Sensitivity

               Study     |	Sen	[95%  Conf. Iterval.]		TP/(TP+FN)  TN/(TN+FP)
--------------------------------------------------------------------------------------------
Smith (2017)             |	0,888     0,797   - 0,947   		71/80	421/453
Sai-Giridhar (2016)      |	0,918     0,886   - 0,943   		356/388	1372/1421
Al-Ramadhani (2013)      |	0,949     0,827   - 0,994   		37/39	111/115
--------------------------------------------------------------------------------------------
          Pooled Sen     |	0,915     0,887   - 0,938   
--------------------------------------------------------------------------------------------
Heterogeneity chi-squared = 1,40 (d.f.= 2) p = 0,496
Inconsistency (I-square) = 0,0 %
No. studies =  3.
Filter ON (Method = 4 )
Add 1/2 to all cells of the studies with zero 


Summary Specificity

               Study     |	Spe	[95%  Conf. Iterval.]		TP/(TP+FN)  TN/(TN+FP)
--------------------------------------------------------------------------------------------
Smith (2017)             |	0,929     0,902   - 0,951   		71/80	421/453
Sai-Giridhar (2016)      |	0,966     0,955   - 0,974   		356/388	1372/1421
Al-Ramadhani (2013)      |	0,965     0,913   - 0,990   		37/39	111/115
--------------------------------------------------------------------------------------------
          Pooled Spe     |	0,957     0,947   - 0,966   
--------------------------------------------------------------------------------------------
Heterogeneity chi-squared = 9,97 (d.f.= 2) p = 0,007
Inconsistency (I-square) = 79,9 %
No. studies =  3.
Filter ON (Method = 4 )
Add 1/2 to all cells of the studies with zero 


Summary Positive Likelihood Ratio (Random effects model)

               Study     |	LR+   	[95%  Conf. Iterval.]		% Weight
--------------------------------------------------------------------------------------------
Smith (2017)             |	12,564    8,916   - 17,704  		38,50
Sai-Giridhar (2016)      |	26,608    20,176  - 35,092  		40,12
Al-Ramadhani (2013)      |	27,276    10,386  - 71,632  		21,38
--------------------------------------------------------------------------------------------
 (REM) pooled LR+        |	20,038    10,716  - 37,469  
--------------------------------------------------------------------------------------------
Heterogeneity chi-squared = 12,67 (d.f.= 2) p = 0,002
Inconsistency (I-square) = 84,2 %
Estimate of between-study variance (Tau-squared) = 0,2343  
No. studies =  3.
Filter ON (Method = 4 )
Add 1/2 to all cells of the studies with zero 


Summary Negative Likelihood Ratio (Random effects model)

               Study     |	LR-   	[95%  Conf. Iterval.]		% Weight
--------------------------------------------------------------------------------------------
Smith (2017)             |	0,121     0,065   - 0,224   		21,50
Sai-Giridhar (2016)      |	0,085     0,061   - 0,119   		74,02
Al-Ramadhani (2013)      |	0,053     0,014   - 0,205   		4,48
--------------------------------------------------------------------------------------------
 (REM) pooled LR-        |	0,090     0,068   - 0,120   
--------------------------------------------------------------------------------------------
Heterogeneity chi-squared = 1,58 (d.f.= 2) p = 0,454
Inconsistency (I-square) = 0,0 %
Estimate of between-study variance (Tau-squared) = 0,0000  
No. studies =  3.
Filter ON (Method = 4 )
Add 1/2 to all cells of the studies with zero 


Summary Diagnostic Odds Ratio (Random effects model)

               Study     |	DOR   	[95%  Conf. Iterval.]		% Weight
--------------------------------------------------------------------------------------------
Smith (2017)             |	103,79    47,525  - 226,66  		37,04
Sai-Giridhar (2016)      |	311,50    196,55  - 493,67  		45,70
Al-Ramadhani (2013)      |	513,38    90,317  - 2918,1  		17,26
--------------------------------------------------------------------------------------------
 (REM) pooled DOR        |	225,99    93,521  - 546,10  
--------------------------------------------------------------------------------------------
Heterogeneity chi-squared = 6,39 (d.f.= 2) p = 0,041
Inconsistency (I-square) = 68,7 %
Estimate of between-study variance (Tau-squared) = 0,3882  
No. studies =  3.
Filter ON (Method = 4 )
Add 1/2 to all cells of the studies with zero
