# Supplementary figures and images for: Detection of mammagloblin by RT-PCR as a biomarker for lymph node metastasis in breast cancer patients: A systematic review and meta-analysis
Source: PLoS One. 2019 May 23;14(5):e0216989. doi: 10.1371/journal.pone.0216989 (PMC6532868; doi:10.1371/journal.pone.0216989)

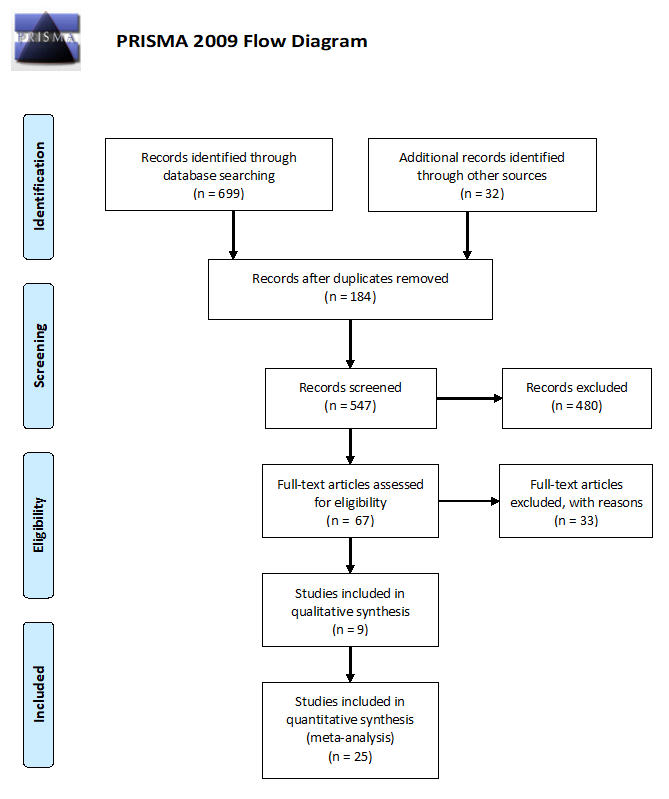

Supplement: S1 Fig — (TIF) [file pone.0216989.s004.tif]
